# Molecular and biological activities of metal oxide-modified bioactive glass

**DOI:** 10.1038/s41598-023-37017-z

**Published:** 2023-06-30

**Authors:** Taha M. Tiama, Hanan Elhaes, Medhat A. Ibrahim, Ahmed Refaat, Mohamed A. M. El-Mansy, Noha M. Sabry

**Affiliations:** 1 Department of Basic Sciences, October High Institute of Engineering & Technology-OHI, 6th of October City, Giza, Egypt; 2 Physics Department, Faculty of Women for Arts, Science and Education, Ain Shams University, Cairo, 11757 Egypt; 3 Molecular Spectroscopy and Modeling Unit, Spectroscopy Department, National Research Centre, 33 El-Bohouth St., Dokki, Giza, 12622 Egypt; 4 Molecular Modeling Simulation Lab, Physics Department, Faculty of Education, Ain Shams University, Roxy, Cairo, Egypt; 5 Water Pollution Research Department, Environment and Climate Change Research Institute, National Research Centre, 33 El-Bohouth St., Dokki, Giza, 12622 Egypt

**Keywords:** Biophysics, Computational biology and bioinformatics

## Abstract

Bioactive glass (BG) was prepared by sol–gel method following the composition 60-($x$) SiO_2_.34CaO.6P_2_O_5_, where *x* = 10 (FeO, CuO, ZnO or GeO). Samples were then studied with FTIR. Biological activities of the studied samples were processed with antibacterial test. Model molecules for different glass compositions were built and calculated with density functional theory at B3LYP/6-31 g(d) level. Some important parameters such as total dipole moment (TDM), HOMO/LUMO band gap energy (ΔE), and molecular electrostatic potential beside infrared spectra were calculated. Modeling data indicated that P_4_O_10_ vibrational characteristics are enhanced by the addition of SiO_2_.CaO due to electron rush resonating along whole crystal. FTIR results confirmed that the addition of ZnO to P_4_O_10_.SiO_2_.CaO significantly impacted the vibrational characteristics, unlike the other alternatives CuO, FeO and GeO that caused a smaller change in spectral indexing. The obtained values of TDM and ΔE indicated that P_4_O_10_.SiO_2_.CaO doped with ZnO is the most reactive composition. All the prepared BG composites showed antibacterial activity against three different pathogenic bacterial strains, with ZnO-doped BG demonstrating the highest antibacterial activity, confirming the molecular modeling calculations.

## Introduction

Recently, phosphate-based glasses have gained a lot of interest as local delivery systems for antimicrobial metal ion delivery due to their ability to dissolve at a constant rate, possessing a non-toxic nature, and having the ability to reach directly to the site of interest. This has led to their use for the development of new antimicrobial agents. Microbes in light of the rapid and continuous development of strains that are resistant to traditional drugs and antibiotics. When cobalt oxide, copper oxide and zinc oxide were added to phosphate glass (5 mg/ml), it showed a strong antimicrobial activity against gram-positive and gram-negative bacteria^1^. Phosphate bioactive glasses doped with metal oxides (MO) have been widely investigated for their antimicrobial efficacy against a range of clinically significant microorganisms^2^. Bioactive glasses (BG) can be used to improve general health because they reduce the risk of bone and joint infections as antimicrobial biomaterials that are used in medical implants^3^. Recently, there has been much interest in focusing on MO nanoparticles because of their numerous applications and as bactericides^4,5^. Because of their abundance, good thermal stability, in addition to low biological toxicity and biodegradability, they are used in a wide variety of applications^6^. When iron oxide (Fe_2_O_3_) is added to bioglass, it increases the density of the crosslinking, and makes it more durable by greatly reducing the rate of decomposition. It can be used to produce fibers of different diameters^2,7^. For example, it has been found that phosphate-containing glass fibers (PGF) supplemented with Fe_2_O_3_ have applications such as being used as cell-delivery compounds for muscle stem cells in order to replace damaged or diseased muscles, and their biocompatibility can be assessed by using other types of cells^8^. When using BG containing Li_2_O-Fe_2_O_3_ and studying the biological activity in the laboratory, the effect of these materials was noticed, as they represented new magnetic biomaterials used as a treatment for cancer with high temperature^9^. Compared with the iron-free glasses, Fe_2_O_3_-doped mesoporous BGs had the ability to increase the standard rate constant of Electro-Fenton's reaction up to 38.44, thus having the ability to produce reactive oxygen species, and can, thus, be very valuable in cancer therapy strategies^10^. It is well known that zinc is essential for all living organisms due to its role in many cellular processes. In addition, there are more than 200 enzymes that need zinc as a cofactor in order to carry out metabolic processes^11^. It was found that there is an antimicrobial efficacy of glass based on phosphate saturated with zinc in the treatment of urinary tract infections^12^. Phosphate glasses doped with zinc oxide at 5 mol% showed promising results for reducing antimicrobial resistance and host cell toxicity^1^. Cu-/Zn-doped borate mesoporous BGs prepared using cost-effective method showed great potential for wound healing/skin tissue engineering applications, associated with excellent antibacterial activity against *Pseudomonas aeruginosa*^13^. Metal ions can be present in the composition of BG (SiO_2_.P_2_O_5_.CaO) prepared by sol–gel method^14^. It was found that copper ions can be easily released from solid particles compared to ions of heavy elements. It can be inferred from this that the release of vital metal ions from the molecules confirms their antimicrobial effect^14^. It was found from the description of copper added to phosphate glass that it has antibacterial properties^8^. This was assessed by studying the effect of CuO and observing the speed of fiber withdrawal on the properties of the glass using fast differential scanning calorimetry (DSC), X-ray diffraction (XRD) and differential thermal analysis (DTA)^7^. The effect of increasing copper content in phosphate-based glass based on Na_2_O.CaO.P_2_O_5_-doped system on the viability of biofilm in vitro was studied^7,8^. Hollow BG nanoparticles enriched with copper and danofloxacin significantly decreased bacterial growth in sessile, planktonic and biofilm states^15^. Characterization of newly developed phosphate-based glass was done with investigating the effect of germanium (Ge) on the structure of SiO_2_.ZnO.CaO.SrO.P_2_O_5_ glass and studying the subsequent effect on the formation of glass polyalkene cements and solubility, as well as bioactivity^16^. Phosphate-based glass with Ge additive (GeO_2_) was developed to enhance nuclear radiation-shielding behaviors and mechanical properties^17^. Owing to the antimicrobial activity of Ge, GeO_2_ was used to enhance the antibacterial properties of silicocarnotite bioceramic, where results showed effective antibacterial activity against Escherichia coli and Staphylococcus aureus^18^.

Molecular modeling with different levels of theory is the most accurate method for predicting structural changes for given model molecules in response to their surrounding chemical environments^19^. It enables researchers to calculate infrared (IR) and Raman spectra with considerable precision^20,21^ for a wide range of molecules. It could also predict some important physical parameters such as total dipole moment (TDM) and HOMO/LUMO band gap energy (ΔE)^22,23^. One can also utilize molecular modeling to investigate surface activity in terms of molecular electrostatic potential (MESP)^24^. Such class of computational work could be utilized for small clusters of atoms which can be successfully used also for glasses simulations^25^.

The aim of the present study is to prepare BG by sol–gel method following the composition 60-($x$) SiO_2_-34CaO-6P_2_O_5_ were *x* = 10 (FeO, CuO, ZnO or GeO). Samples were studied with Fourier transform infrared spectroscopy (FTIR) then their biological activities were processed with antibacterial test. Model molecules for the different glass compositions were conducted with density functional theory (DFT) at B3LYP/6-31 g(d) level. Some important parameters such as TDM, molecular electrostatic potential, as well as infrared spectra were calculated.

## Materials and methods

### Calculations details

All the studied models were subjected to quantum mechanical calculations using GAUSSIAN 09^26^ softcode at Molecular Spectroscopy and Modeling Unit, Spectroscopy Department, National Research Centre, Cairo Egypt. DFT at B3LYP/6-31 g(d)^27–29^ level was used to calculate TDM, ΔE, MESP and IR frequencies. Partial density of states (PDOS) plots were generated using GaussSum^30^.

### Materials

BG was synthesized by sol–gel method and the composition of the samples was [60-($x$) SiO_2_.34CaO.6P_2_O_5_] [were *x* = 10, from FeO, CuO, ZnO or GeO] (wt.%), as shown in Table 1. The precursors used for BG were Tetra-Ethyl-Ortho-Silicate (TEOS, Sigma Aldrich, Merck, Germany), Calcium Nitrate Tetrahydrate (Ca(NO_3_)_2_.4H_2_O, Merck, Germany), and Tetri-Ethyl-Phosphate (TEP) (Sigma Aldrich, Merck, Germany). Other chemicals were used such as ammonium hydroxide (NH_4_OH, Merck), nitric acid (Merck, Germany), Ethyl Alcohol and deionized water.

**Table 1:** The compositions of the parent and doped-glasses in wt%. Labels of the glasses are also reported.

Glass	Glass base			Additives wt%			
	SiO_2_	P_2_O_5_	CaO	FeO	GeO	CuO	ZnO
BG (control)	60	6	34	0	0	0	0
BG FeO 10%	50	6	34	10	0	0	0
BG GeO 10%	50	6	34	0	10	0	0
BG CuO 10%	50	6	34	0	0	10	0
BG ZnO 10%	50	6	34	0	0	0	10

### Methods

#### Synthesis of glass powder

Samples were prepared using the sol–gel method reported by Tohamy et al.^31^. The preparation of the gels involved using a quick alkali-mediated sol–gel method. This system consisted of four samples in addition to the control sample. BG was doped with [FeO, CuO, ZnO or GeO] (10 wt%). In the first step, TEOS was dissolved in ethanol/nitric acid solution and deionized H_2_O with continuous stirring for 45 min. Then, calcium nitrate tetrahydrate was added to the solution and stirred for 45 min. TEP was finally added to the solution and stirred for 45 min. After the final addition, the mixture of all reagents was left for 60 min to complete hydrolysis. Ammonia solution of 2 M concentration (a gelation catalyst) was dropped into the mixture. The mixture was then manually agitated with glass rod (as a mechanical stirrer) to prevent the formation of a bulk gel. Finally, each prepared gel was left to dry at 100–120 °C for 2 days and sintered at 600 °C for 2 h in thermal oven.

#### Antibacterial activity of prepared glass composites nanoparticles

The antibacterial activity of BG composites was determined using the paper disc diffusion assay reported earlier^32^. In brief, Tryptic Soy Agar (TSA) plates were prepared and inoculated with a 1 mL cell suspension of each bacterial pathogen separately, including gram-negative bacteria *Pseudomonas aeruginosa* (ATCC 10145) and *Aeromonas hydrophila* strain, and gram-positive bacteria *Staphylococcus aureus* (ATCC 25923), obtained from the Microbial Inoculants Center—MIC, Ain Shams University, Egypt. Each pathogenic bacteria strain's pure culture was inoculated on TSA plates and incubated for 24 h at 37 °C. 4–5 loops from each strand were transferred into culture tubes containing 5 mL of sterile Tryptic Soy Broth (TSB) and incubated for 12 h at 37 °C. Mueller Hinton agar plates were inoculated with a 10^6^ cell/mL microorganism suspension using cotton swabs. Sterile filter paper discs (Whatman^®^ Glass Microfibre filters, 6 mm in diameter) were impregnated with 20 µg/mL of various MO nanoparticle solutions.

## Results and discussions

### Molecular modeling

#### Building model molecules

**Figure 1:**
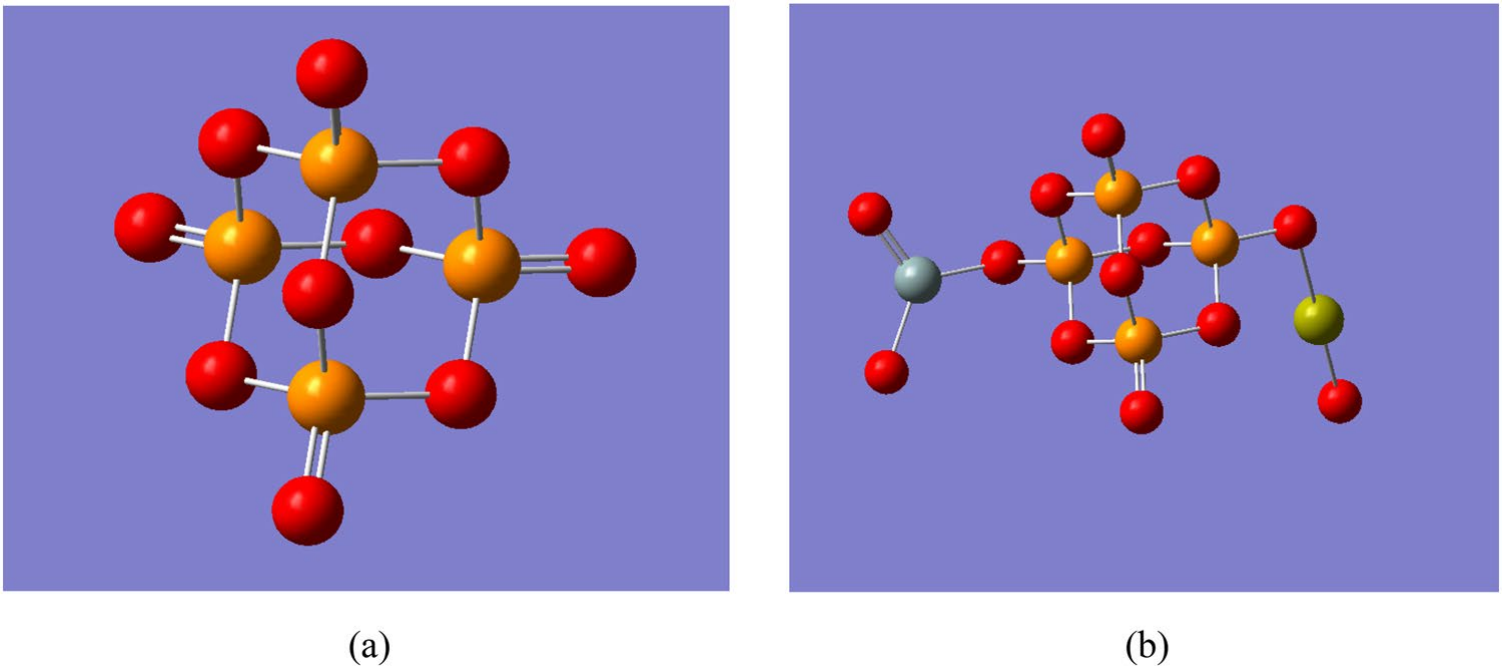
Structure of the main model molecules for phosphate glass; (**a**) P_4_O_10_ unit and (**b**) P_4_O_10_.SiO_2_.CaO unit.

**Figure 2:**
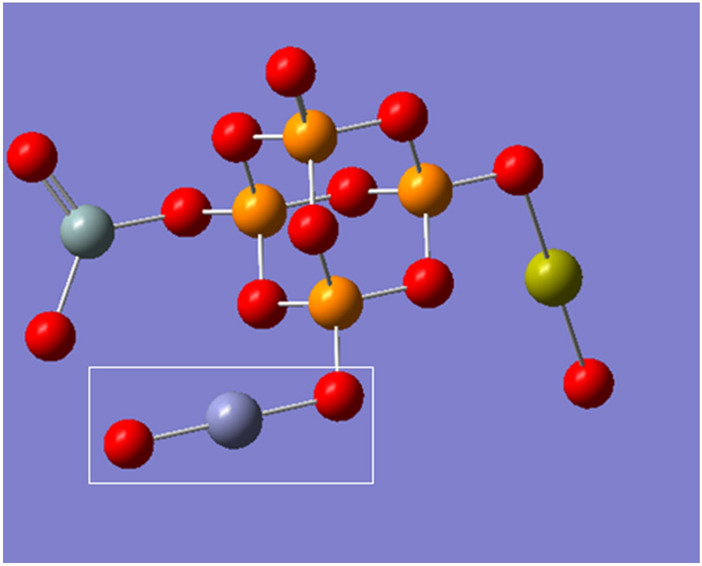
Structure of the main model molecules for phosphate glass doped with metal oxide (CuO; FeO; ZnO and GeO). The position of the metal oxide is marked with rectangle.

Two phosphate molecules were gathered as a unit cell to build a model molecule for phosphate which is termed as P_4_O_10_ and demonstrated in Fig. 1a. Then another model molecule representing phosphate silicate with calcium oxide was built and termed as P_4_O_10_.SiO_2_.CaO, which is demonstrated in Fig. 1b. Using the same route, another four models were built and termed as P_4_O_10_.SiO_2_.CaO.MO where MO is CuO, FeO, ZnO or GeO. The four model molecules are demonstrated in Fig. 2.

Therefore, a total of 6 model molecules were built to study the effect of MO upon P_4_O_10_.SiO_2_.CaO BG. Assignment of the calculated vibrational spectra of the model molecules was done using the same software.

#### Theoretical IR band assignments

**Figure 3:**
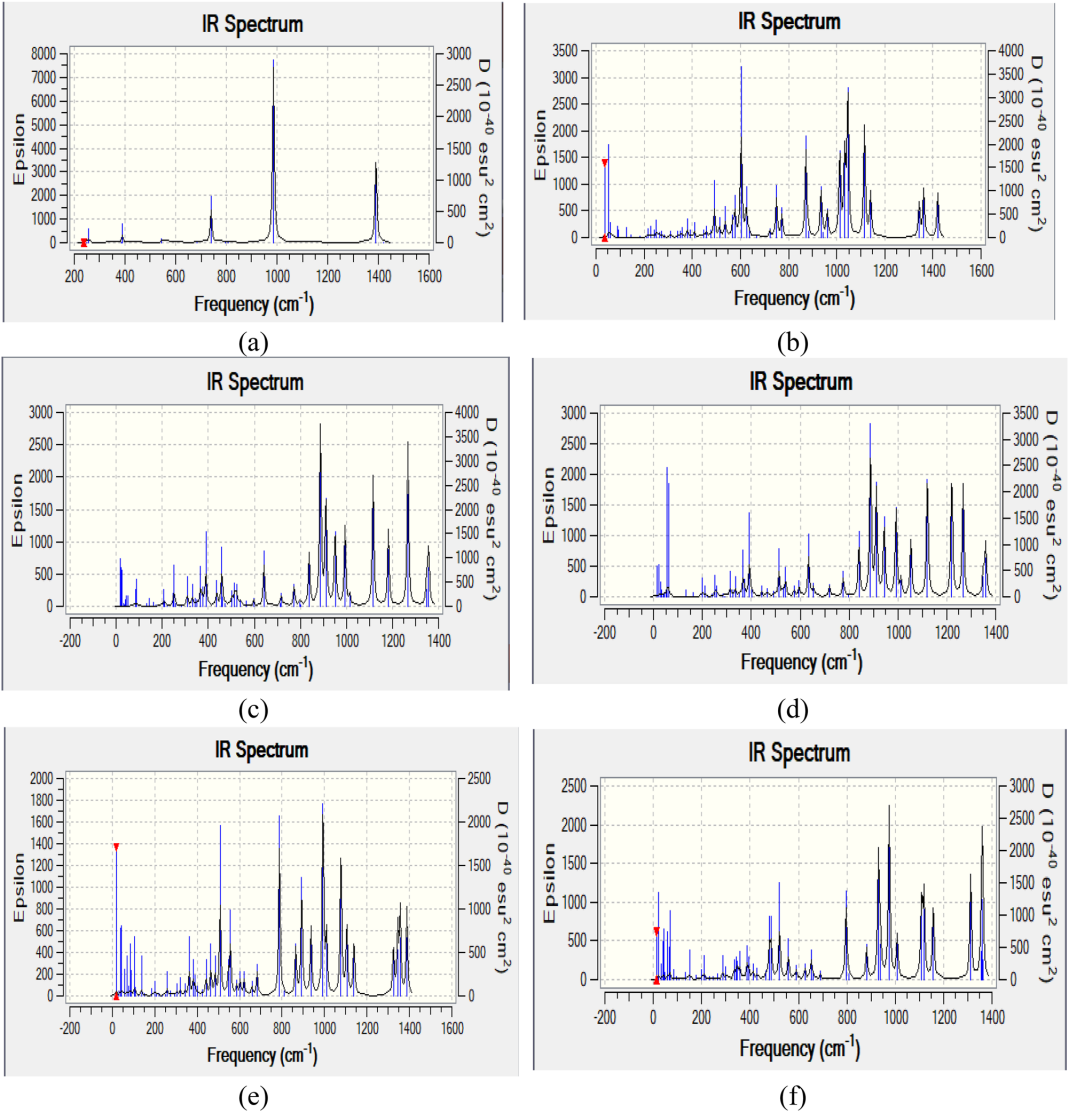
Calculated IR spectra for (**a**) P_4_O_10_; (**b**) P_4_O_10_.SiO_2_.CaO; (**c**) P_4_O_10_.SiO_2_.CaO.CuO; (**d**) P_4_O_10_.SiO_2_.CaO.FeO; (**e**) P_4_O_10_.SiO_2_.CaO.ZnO; and (**f**) P_4_O_10_.SiO_2_.CaO.GeO.

The DFT:B3LYP/6-31 g(d) level was used to optimize then calculating vibrational spectra of the studied 6 model molecules. Figure 3 presents the calculated IR spectra for a- P_4_O_10_; b- P_4_O_10_.SiO_2_.CaO; c- P_4_O_10_.SiO_2_.CaO.CuO; d- P_4_O_10_.SiO_2_.CaO.FeO; e- P_4_O_10_.SiO_2_.CaO.ZnO and f- P_4_O_10_.SiO_2_.CaO.GeO. GaussView software animation was used to visualize the spectra then the assignment is aided by the software.

As indicated in Fig. 3a, for intrinsic P_4_O_10_: Four main modes appeared in the chart (P-O wagging $\sim$ 389 cm^−1^, P–O scissoring $\sim$ 740 cm^−1^, and P–O and P = O stretching at 986 and 1390 cm^−1^, respectively).

For P_4_O_10_.SiO_2_.CaO calculated IR spectrum shown in Fig. 3b: P–O wagging is upshifted to 492 cm^−1^, as well as P-O scissoring $\sim$ 750 cm^−1^. Si–O stretching is centered at 872 cm^−1^, while P-O stretches are triplized via the peaks at 1013, 1033 and 1047 cm^−1^. Si = O Stretch is positioned at 1343 cm^−1^ while P = O stretching appeared at 1362 and 1420 cm^−1^.

Figure 3c demonstrates the calculated IR spectrum for P_4_O_10_.SiO_2_.CaO.CuO: Si–O and P-O wagging vibrations are noticed at 390 and 437 cm^−1^, respectively. Ca–O and P–O scissoring vibrations are noticed at 459 and 642 cm^−1^, respectively. P–O stretching vibrations appeared at 892, 936 and 992 cm^−1^. Si–O and Si = O stretching are seen at 1009 and 1345 cm^−1^, respectively. P = O stretching is assigned at 1357 cm^−1^.

In the calculated IR spectrum of P_4_O_10_.SiO_2_.CaO.FeO shown in Fig. 3d: Si–O and P-O wagging vibrations are noticed at 392 and 513 cm^−1^, respectively. Ca–O and P–O scissoring are detected at 634 and 776 cm^−1^, respectively. P–O stretching vibrations are centered at 840, 887 and 911 cm^−1^. Si–O and Si = O stretching vibrations appeared at 992 and 1350 cm^−1^, respectively. P = O stretch is perceived at 1360 cm^−1^.

In Fig. 3e which depicts the calculated IR spectrum of P_4_O_10_.SiO_2_.CaO.ZnO: P-O wagging and scissoring is uplifted to 508 and 787 cm^−1^, respectively. Zn–O stretching is found at 864 cm^−1^, and P–O stretching vibrations are centered at 892, 936 and 992 cm^−1^. Si–O and Si = O stretching vibrations are seen at 1009 and 1345 cm^−1^. P = O stretching vibrations are noted at 1357 and 1388 cm^−1^.

As indicated in Fig. 3f showing the calculated IR spectrum of P_4_O_10_.SiO_2_.CaO.GeO: Si–O and P-O wagging are noticed at 387 and 487 cm^−1^, respectively. Ca–O and P–O scissoring are detected at 626 and 652 cm^−1^, respectively. P-O stretching vibrations are distinguished at 797, 881, 930 and 974 cm^−1^. Si–O and Si = O stretching vibrations are seen at 1008 and 1361 cm^−1^. P = O stretching is assigned to the band at 1356 cm^−1^.

Correlating the above data, one can conclude that the vibrational characteristics of P_4_O_10_ are enhanced by SiO_2_.CaO additive due to electron rush resonating along whole crystal. Additive ZnO to P_4_O_10_. SiO_2_.CaO highly impacted the vibrational characteristics in contradiction to the other alternatives CuO, FeO and GeO that caused minimal change in spectral indexing.

In order to experimentally verify the above findings, BG was prepared then characterized using FTIR. The experimental approach is needed to verify the molecular modeling data.

#### Molecular electrostatic potential mapping

**Figure 4:**
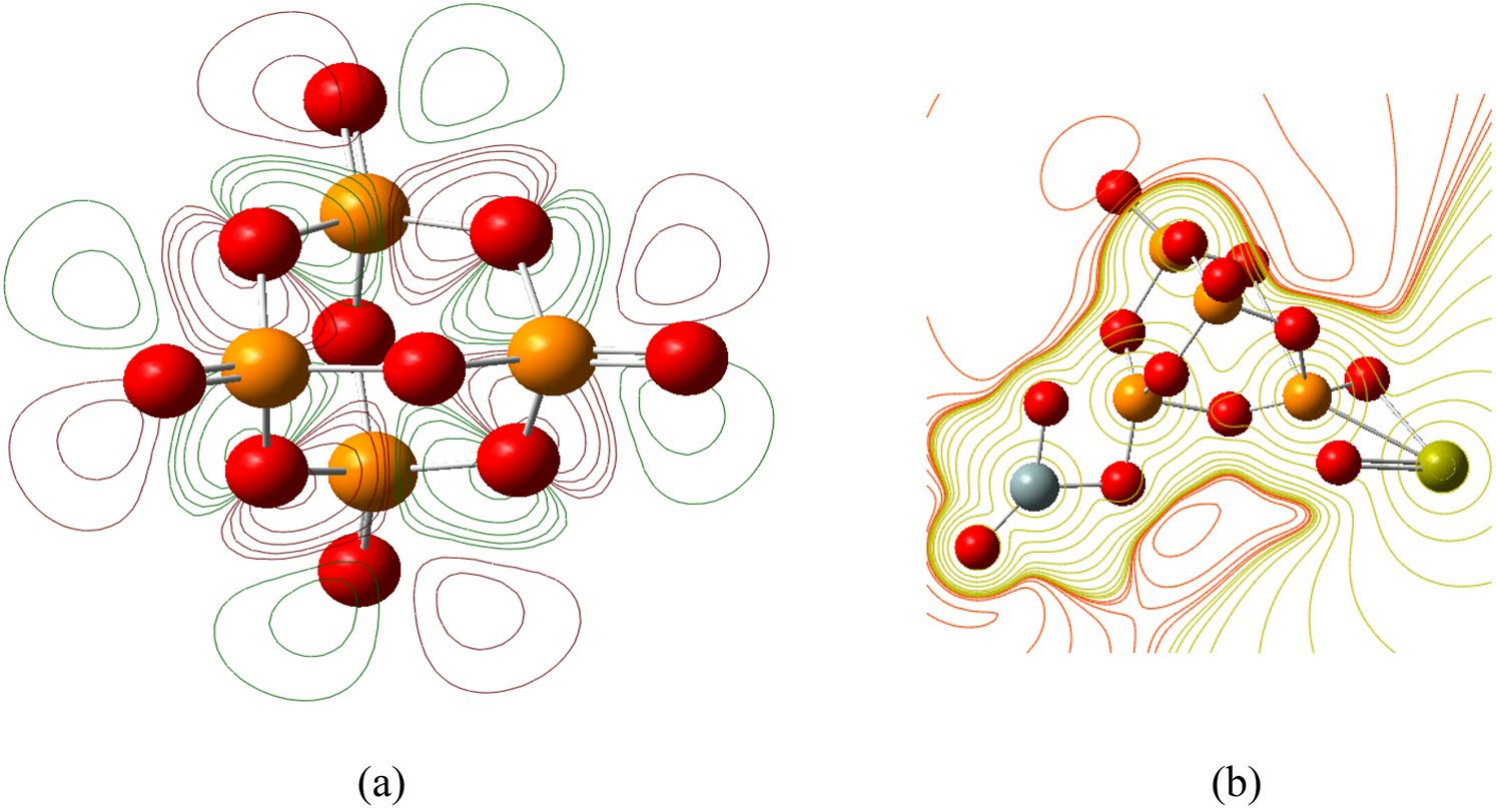
Molecular electrostatic potential maps for the main model molecules for phosphate glass; (**a**) P_4_O_10_ unit; and (**b**) P_4_O_10_.SiO_2_.CaO unit.

A molecular electrostatic potential (MESP) map is useful three-dimensional plot that demonstrates the distribution of electron density (charges) around the molecule and can, thus, be used to predict the charge-related properties of molecules, and to identify the site of electrophilic and nucleophilic attacks^33^. MESP map of a molecule's surface can be easily interpreted in terms of different colors, which are arranged from highest to lowest electron density (electronegativity) in the following order: red > orange > yellow > green > blue. Therefore, the lowest electrostatic potential is found in red regions, whereas the highest electrostatic potential is found in blue. Based on this and as seen in Fig. 4a, the oxygen atoms in the P_4_O_10_ unit displayed higher electron density than phosphorus atoms. In Fig. 4b, significant change took place in the MESP map with the presence of SiO_2_ and CaO with, again, oxygen atoms representing the regions of higher electronegativity.

**Figure 5:**
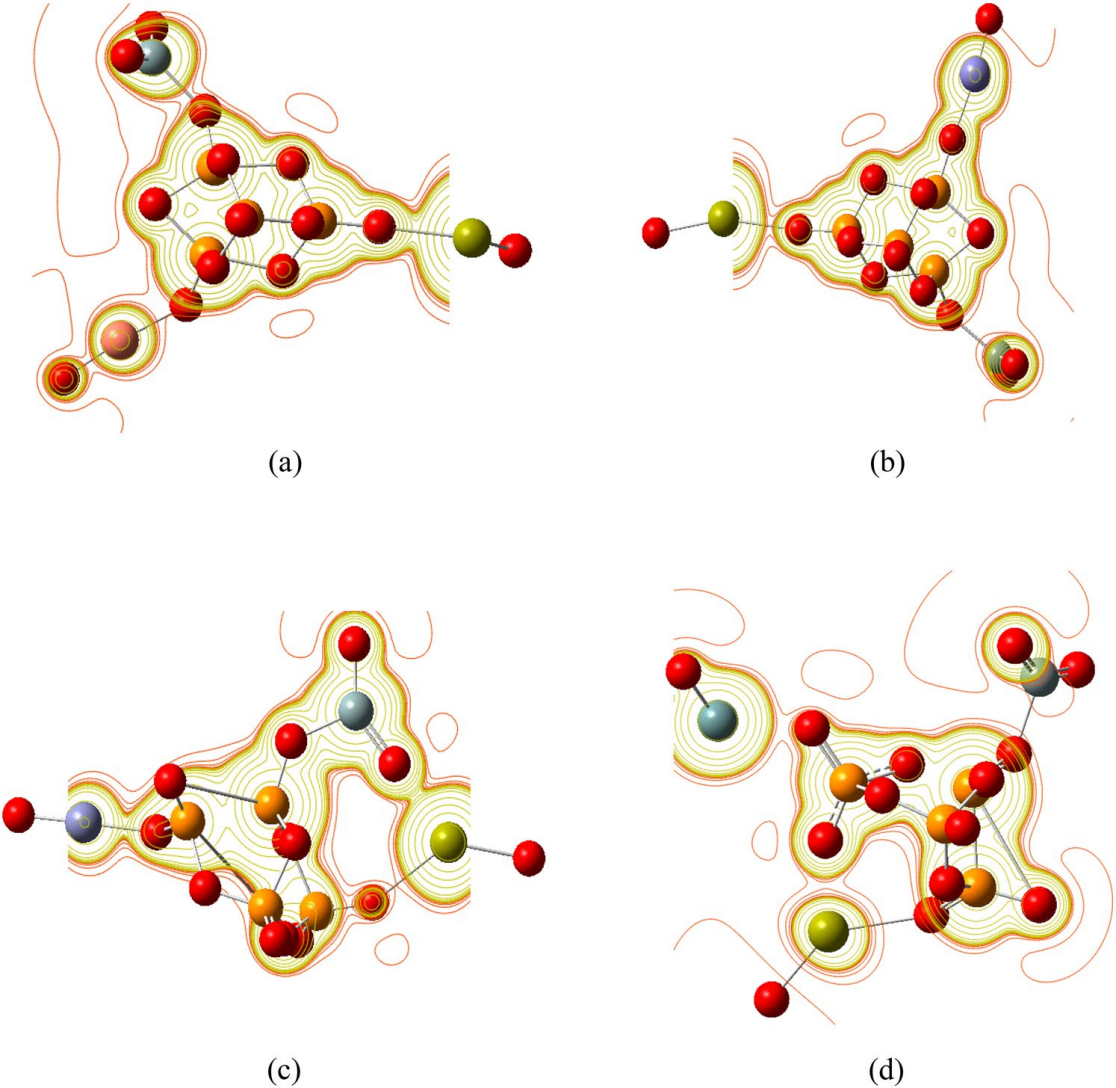
Molecular electrostatic potential maps for the phosphate glass doped with metal oxide (**a**) CuO; (**b**) FeO; (**c**) ZnO and (**d**) GeO.

Similar behavior can be clearly seen in Fig. 5 where CuO, FeO, ZnO and GeO metal oxide dopants resulted in significant change in the electron density distribution and electronegativity on the molecule's surface, thus introducing sites ready for nucleophilic attack and others ready for electrophilic attack.

The bacterial membranes are negatively charged due to the highly electronegative groups on membrane's phospholipids and lipopolysaccharides^34^. Therefore, it has been reported that the membrane may be the site of the antimicrobial activity of cationic metals attracted to it via electrostatic attraction^34–36^. It can, thus, be concluded that differences in electronegativity play a major role and has a direct effect on antimicrobial activity^37^.

#### Total dipole moment and HOMO/LUMO band gap energy

The dipole moments are highly sensitive even to small errors and are, therefore, an efficient check for the quality of computations, and in describing the overall electron density properties^38^. TDM is used to detect the nature of reactivity and impurity atoms effect of system, and it is well established in several studies that TDM is closely related to reactivity in such a way that the higher the TDM, the higher the reactivity^38–40^.

Band gap energy is the difference between the highest occupied molecular orbital (HOMO) and lowest unoccupied molecular orbital (LUMO) in the system. ΔE has been used as a simple indicator of the molecular chemical stability and could indicate the affinity pattern of the molecule^41^.

The calculated values of TDM and ΔE are listed in Table 2. The values demonstrated significant increase in the value of TDM in BG, measuring 27.1994 Debye, compared to P_4_O_10_ for which the TDM was zero. ΔE was well correlated with TDM, showing significant decrease in its value to 1.7129 eV for BG, compared to 8.0268 eV for P_4_O_10_. This result indicates significant increase in the reactivity of the BG.

**Table 2:** Calculated total dipole moment TDM as Debye and ΔE as eV.

Structure	TDM	ΔE
P_4_O_10_	0.0000	8.0268
P_4_O_10_.SiO_2_.CaO	27.1994	1.7129
P_4_O_10_.SiO_2_.CaO.CuO	4.4413	1.7004
P_4_O_10_.SiO_2_.CaO.FeO	5.4207	1.5186
P_4_O_10_.SiO_2_.CaO.ZnO	9.4401	0.9660
P_4_O_10_.SiO_2_.CaO.GeO	8.2151	1.2871

The results of TDM and ΔE energy after doping of BG with different MO indicated that all BG.MO structures experienced significant decrease in their TDM compared to BG, with ΔE also showing decrease in its value for all BG.MO structures. However, comparing the values of TDM and ΔE of the four BG.MO structures with one another, it is clear that BG.ZnO is the most reactive structure owing to its highest TDM and lowest ΔE. In addition, it has been reported that ZnO nanoparticles, interestingly, contain a positive charge in water suspensions^42^. Correlating TDM and ΔE results with those of MESP mapping, and since the total bacterial charge is negative because of the negatively charged bacterial membranes, it can be concluded that BG.ZnO has a greater affinity to create electrostatic forces as a powerful bond with the bacterial surface, thus providing a stronger antibacterial effect^43^, which is confirmed by the assessment of antibacterial activity of the different glass composites, as later presented in Section “Assessment of antibacterial activity”.

#### Partial density of states

**Figure 6:**
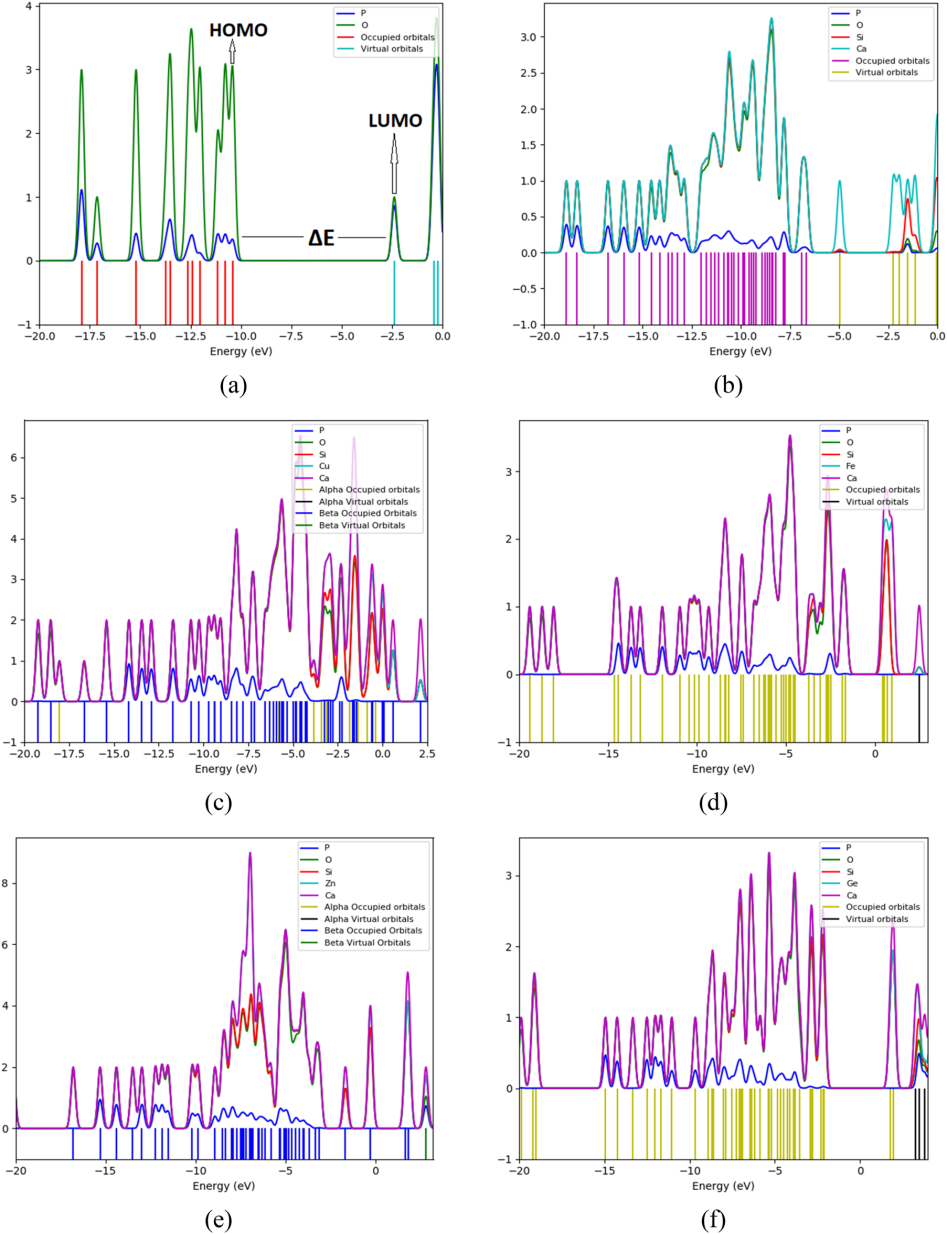
PDOS plots of (**a**) P_4_O_10_; (**b**) P_4_O_10_.SiO_2_.CaO; (**c**) P_4_O_10_.SiO_2_.CaO.CuO; (**d**) P_4_O_10_.SiO_2_.CaO.FeO; (**e**) P_4_O_10_.SiO_2_.CaO.ZnO; and (**f**) P_4_O_10_.SiO_2_.CaO.GeO.

In order to thoroughly determine the effect of the MOs on the electronic structure/band gap of BG and the contribution of each metal, PDOS plots are depicted in Fig. 6. The wide ΔE of P_4_O_10_ molecule, mentioned in Table 2, which reflects its low reactivity compared to the other structures, is clearly demonstrated in Fig. 6a. The atomic orbitals contribution of P and O in the PDOS plot of P_4_O_10_ reflects that the atomic orbitals of both P and O contributed to the valence states, with the 2p atomic orbitals of O demonstrating higher contribution for the HOMO than the 3p atomic orbitals of P. Whereas, the LUMO of the conduction band indicates almost equal contribution by P and O atomic orbitals. In Fig. 6b depicting the PDOS of P_4_O_10_.SiO_2_.CaO, ΔE showed significant decrease compared to that shown in P_4_O_10_ plot. Also HOMO showed almost equal contributions of the atomic orbitals of O, Si and Ca, with very minimal contribution of the atomic orbitals of P, while LUMO is clearly dominated by the contribution of the atomic orbitals of Ca.

The PDOS plot of BG CuO shown in Fig. 6c, the atomic orbitals of Ca contributed the most to HOMO, with lower and very close contributions of the atomic orbitals of O, Si and Cu, and no contribution of the atomic orbitals of P. The atomic orbitals contribution in LUMO showed the same behaviour. In Fig. 6d, the PDOS plot of BG FeO showed that the atomic orbitals of O and Si had close contribution to HOMO, with higher contribution by 3d and 4 s atomic orbitals of Fe and the highest contribution was by the 4 s atomic orbital of Ca.

In the PDOS plot of BG ZnO demonstrated Fig. 6e, the narrowest ΔE is clearly visualized, reflecting the highest reactivity of this structure compared with the other structures. The highest contribution to HOMO is offered by the atomic orbitals of Ca followed by closer contributions of those of Zn, O and Si, with no contribution from the atomic orbitals of P. The highest contribution for LUMO was from the atomic orbitals of Ca, followed by very close contributions from the atomic orbitals of Zn and Si, followed by O and finally P. The final PDOS plot presented in Fig. 6f for BG GeO indicated that the highest contribution for HOMO is given by the atomic orbitals of Ca, followed by very close contributions of the atomic orbitals of Ge, O and Si, and no contribution from P, and LUMO had the highest contribution from Ca, followed by Ge, then Si, O and the lowest contribution was from the atomic orbitals of P.

### Experimental FTIR band assignments

**Figure 7:**
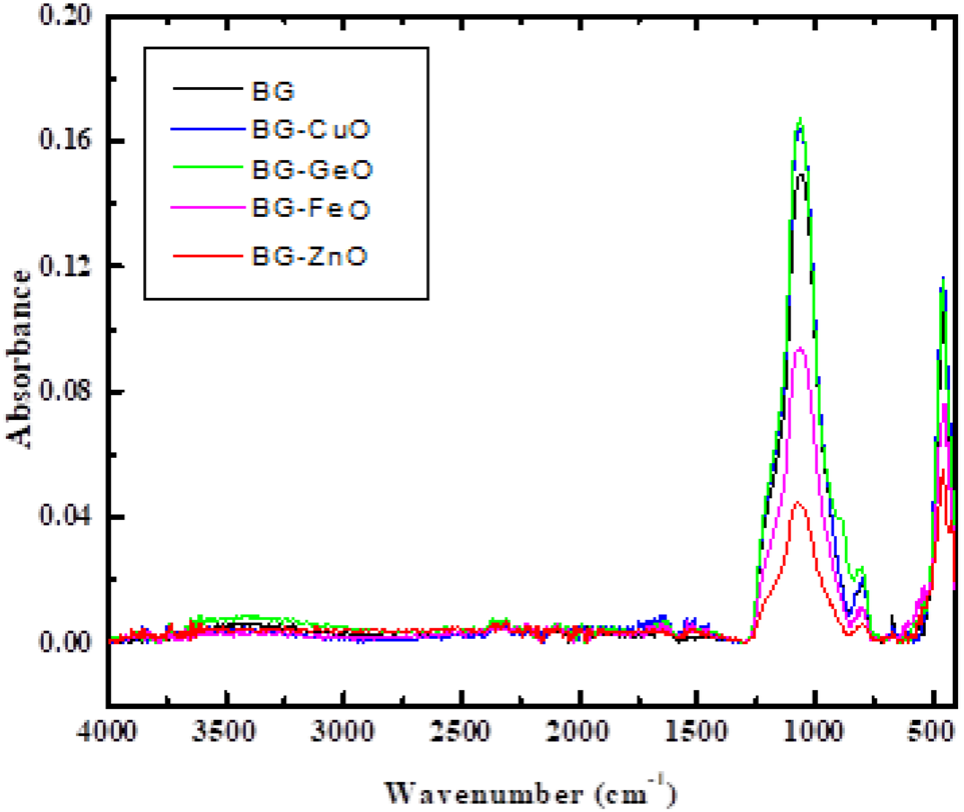
FTIR absorption spectra for BG and BG Modified with four metal oxides (CuO; GeO; FeO and ZnO).

Figure 7 demonstrates the FTIR absorption spectra for BG and BG modified with four metal oxides (CuO, GeO, FeO and ZnO).

In the spectrum of BG: P–O wagging is centered at 456 cm^−1^ and P-O scissoring is centered at $\sim$ 662 cm^−1^. Si–O stretching is noted at 796 cm^−1^ while P–O stretching is noted at 1059 cm^−1^. BG/P-O scissoring vibrations were upshifted to 561 cm^−1^ by additive ZnO, while downshifted to 452 cm^−1^ by additive CuO, to 454 cm^−1^ by GeO, and to 452 cm^−1^ by FeO. BG/Si–O stretching vibrations were unaffected by additive FeO and appeared at 796 cm^−1^. Whereas, it was enhanced by additives CuO (803 cm^−1^) and GeO (887 cm^−1^), and reduced by ZnO (972 cm^−1^). BG/P-O wagging vibrations experienced a gradual upshift by additives ZnO (664 cm^−1^), CuO (666 cm^−1^) and GeO (794 cm^−1^) and a sudden down grading by additive Fe_2_O (628 cm^−1^). Also, BG/P-O stretching vibrations were significantly affected by additives CuO and GeO (1066 cm^−1^), FeO (1079 cm^−1^), and ZnO (1082 cm^−1^). Results indicated that a perfect agreement between theoretical and experimental data is established, reflecting high accuracy of computations.

### Assessment of antibacterial activity

BG has been strongly advocated as a potential replacement for the graft materials currently in use^44^. According to reports, borate-based biomaterials were used at the infection site because of their antibacterial properties^45^.

**Figure 8:**
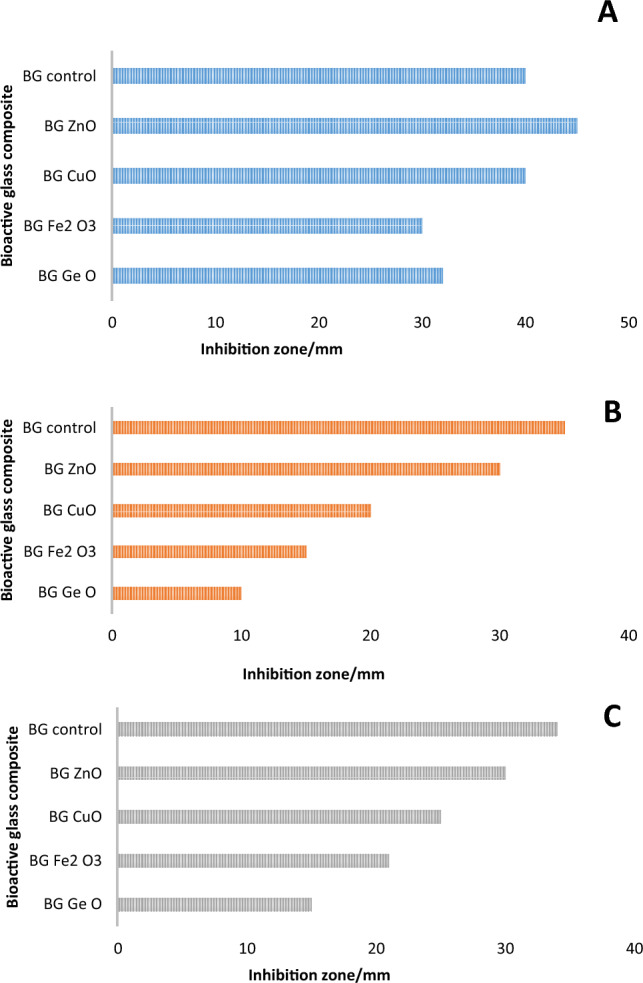
Antibacterial activity of BG and BG/metal oxide nanocomposites against (**A**) *S. aureus* (**B**) *P. aeruginosa*, and (**C**) *A. hydrophila.*

Antibacterial activity was found in all of the tested composites as shown in Fig. 8 and Table 3. In the case of *S. aureus* shown in Fig. 8a, it was found that coating the glass composite particles with ZnO nanoparticles increased the antibacterial activity, being the maximum among the used MO by creating an inhibition zone of 50 mm. Coating with FeO, on the other hand, showed the minium activity, producing a 30 mm inhibition zone, which also reflects lower antibacterial activity towards *S. aureus* than BG control which created a 40 mm inhibition zone. Therefore, comparing the antibacterial activity of MO-coated BG based on the diameter of the inhibition zones shown in Table 3 confirmed that BG.ZnO demonstrated the highest activity, followed by BG control and BG.CuO (40 mm each), BG.GeO. (32 mm), and BG.FeO (30 mm).

**Table 3:** Inhibition zones of 200 µg/ml of each glass composite against three different bacterial pathogens.

Pathogens	Inhibition zone (mm)				
	BG control	BG.ZnO	BG.CuO	BG.FeO	BG.GeO
*S. aureus*	40	50	40	30	32
*P. aeruginosa*	35	30	20	15	10
*A. hydrophila*	34	30	15	25	21

The inhibition zones displayed in Fig. 8b also confirmed antibacterial activity against *P. aeruginosa*, where BG control and BG.ZnzO showed the highest antibacterial activity with inhibition zones of 35 and 30 mm, respectively. Furthermore, BG.CuO had higher antibacterial activity than BG.FeO, and BG.GeO which showed the lowest antibacterial activity.

**Figure 9:**
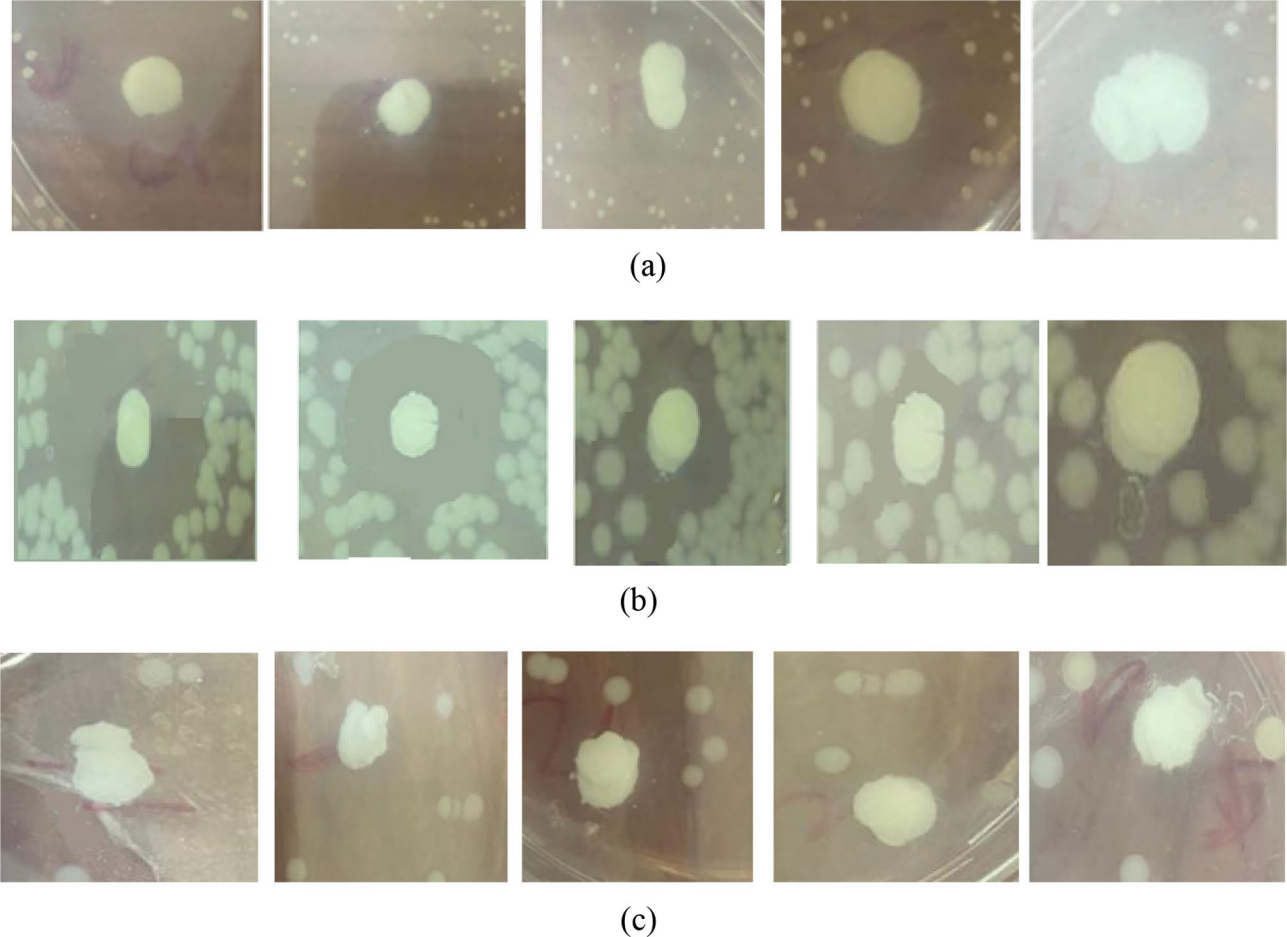
Inhibition zones of BG and different BG/metal oxide nanocomposites for (**a**) *S. aureus*, (**b**) *P. aeruginosa*, and (**c**) *A. hydrophila*.

In the case of *A. hydrophila,* all BG composition again showed antibacterial activity as shown in Fig. 8c. Similarly, BG control and BG.ZnO demonstrated the highest antibacterial activity based on their inhibition zones listed in Table 3. BG.FeO presented lower antibacterial activity followed by BG.GeO, then BG.CuO which present the lowest antibacterial activity against *A. hydrophila*. Composites inhibited the bacterial strains tested. This again could be due to the effect produced by the presence of boron and silicon ions present in the base composition of BG, as well as the additional effect posed by the MOs. The inhibition zones in the three bacterial strains *S. aureus*, *P. aeruginosa,* and *A. hydrophila* created by the different BG composite glass are demonstrated in Fig. 9.

## Conclusion

Phosphate-based (P_4_O_10_.SiO_2_.CaO) bioactive glass (BG control) was synthesized using sol–gel method and doped with 10 wt% of four different metal oxides (MOs) individually, namely FeO, CuO, ZnO or GeO. Experimental FTIR spectra of BG and metal oxide-doped BG confirmed the proper formation of BG matrix, and enhancement of the vibrational characteristics of P_4_O_10_ by SiO_2_.CaO additive. Doping of P_4_O_10_.SiO_2_.CaO with ZnO also highly impacted the vibrational characteristics, while doping with CuO, FeO and GeO demonstrated only minimal change in vibrational characteristic.

Theoretical infrared spectra, electronic properties and molecular electrostatic potential maps (MESP) were studied using DFT molecular modeling calculations at B3LYP/6-31 g(d) level. Theoretical infrared spectral were in perfect agreement with experimental data, reflecting high accuracy of computations. The reactivity of the different glass compositions was evaluated in terms of the electronic properties represented by total dipole moment (TDM), HOMO/LUMO band gap energy (ΔE) and MESP. All BG.MO structures demonstrated significant decrease in their TDM values compared to BG control, with ΔE also showing decrease in its value for all BG.MO composites. Within the four BG.MO composites, BG.ZnO is the most reactive structure owing to its highest TDM and lowest ΔE. MESP confirmed that oxygen atoms in the P_4_O_10_ unit has higher electron density than phosphorus atoms. Oxygen atoms continued to show higher electronegativity with the presence of SiO_2_ and CaO. Doping with CuO, FeO, ZnO and GeO resulted in significant change in the electron density distribution and electronegativity on the molecule's surface, introducing sites ready for nucleophilic attack and others ready for electrophilic attack.

The antibacterial activity of the BG control and the four BG.MO composites was assessed against *S. aureus*, *P. aeruginosa* and *A. hydrophila* pathogenic bacterial strains. All glass compositions showed antibacterial activity against the three pathogens, with BG.ZnO showing the highest antibacterial activity among the four BG.MO composites. This result is well correlated with molecular modeling results of reactivity and MESP owing to the highest reactivity of BG.ZnO, and the positive charge of ZnO nanoparticles in water suspensions, thus having a greater affinity to create electrostatic forces as a powerful bond with the negatively charged bacterial surface, and providing a stronger antibacterial effect.

## Data Availability

The data will be available upon request. Contact Medhat A. Ibrahim: medahmed6@yahoo.com.

## References

[1] Raja FNS, Worthington T, de Souza LPL, Hanaei SB, Martin RA (2022). Synergistic antimicrobial metal oxide-doped phosphate glasses; a potential strategy to reduce antimicrobial resistance and host cell toxicity. ACS Biomater. Sci. Eng..

[2] Miola M (2018). Copper-doped bioactive glass as filler for PMMA-based bone cements: Morphological, mechanical, reactivity, and preliminary antibacterial characterization. Materials.

[3] Maany DA, Alrashidy ZM, Abdel Ghany NA, Abdel-Fattah WI (2019). Comparative antibacterial study between bioactive glasses and vancomycin hydrochloride against *Staphylococcus*
*aureus*, *Escherichia*
*coli* and *Pseudomonas*
*aeruginosa*. Egypt. Pharmaceut. J..

[4] Saber S (2018). Annealing study of electrodeposited CuInSe_2_ and CuInS_2_ thin films. Opt. Quant. Electron..

[5] El Nahrawy AM, Abou Hammad AB, Bakr AM, Shaheen TI, Mansour AM (2020). Sol–gel synthesis and physical characterization of high impact polystyrene nanocomposites based on Fe_2_O_3_ doped with ZnO. Appl. Phys. A.

[6] El Nahrawy AM, Hammad ABA, Youssef AM, Mansour AM, Othman AM (2019). Thermal, dielectric and antimicrobial properties of polystyrene-assisted/ITO: Cu nanocomposites. Appl. Phys. A.

[7] Abou Neel EA, Ahmed I, Pratten J, Nazhat SN, Knowles JC (2005). Characterisation of antibacterial copper releasing degradable phosphate glass fibres. Biomaterials.

[8] Mulligan AM, Wilson M, Knowles JC (2003). The effect of increasing copper content in phosphate-based glasses on biofilms of *Streptococcus sanguis*. Biomaterials.

[9] Arabyazdi S, Yazdanpanah A, Hamedani AA, Ramedani A, Moztarzadeh F (2019). Synthesis and characterization of CaO-P_2_O_5_-SiO_2_-Li_2_O-Fe_2_O_3_ bioactive glasses: The effect of Li_2_O-Fe_2_O_3_ content on the structure and in-vitro bioactivity. J. Non-Cryst. Solids.

[10] Kermani F (2022). Iron (Fe)-doped mesoporous 45S5 bioactive glasses: Implications for cancer therapy. Transl. Oncol..

[11] MacDonald RS (2000). The role of zinc in growth and cell proliferation. J. Nutr..

[12] Raja FNS, Worthington T, Isaacs MA, Rana KS, Martin RA (2019). The antimicrobial efficacy of zinc doped phosphate-based glass for treating catheter associated urinary tract infections. Mater. Sci. Eng. C.

[13] Kermani F (2023). Zinc- and copper-doped mesoporous borate bioactive glasses: Promising additives for potential use in skin wound healing applications. Int. J. Mol. Sci..

[14] Palza H (2013). Designing antimicrobial bioactive glass materials with embedded metal ions synthesized by the sol–gel method. Mater. Sci. Eng. C.

[15] Jiménez-Holguín J, Sánchez-Salcedo S, Cicuéndez M, Vallet-Regí M, Salinas AJ (2022). Cu-doped hollow bioactive glass nanoparticles for bone infection treatment. Pharmaceutics.

[16] Mokhtari S (2019). Investigating the effect of germanium on the structure of SiO_2_-ZnO-CaO-SrO-P_2_O_5_ glasses and the subsequent influence on glass polyalkenoate cement formation, solubility and bioactivity. Mater. Sci. Eng. C.

[17] Saddeek YB (2020). Alkaline phosphate glasses and synergistic impact of germanium oxide (GeO_2_) additive: Mechanical and nuclear radiation shielding behaviors. Ceram. Int..

[18] Ji Y, Yang S, Sun J, Ning C (2023). Realizing both antibacterial activity and cytocompatibility in silicocarnotite bioceramic via germanium incorporation. J. Funct. Biomater..

[19] Stoch P, Stoch A, Ciecinska M, Krakowiak I, Sitarz M (2016). Structure of phosphate and iron-phosphate glasses by DFT calculations and FTIR/Raman spectroscopy. J. Non-Cryst. Solids.

[20] Labet V, Colomban P (2013). Vibrational properties of silicates: A cluster model able to reproduce the effect of "SiO_4_" polymerization on Raman intensities. J. Non-Cryst. Solids.

[21] Wallace S, Lambrakos SG, Shabaev A, Massa L (2022). On using DFT to construct an IR spectrum database for PFAS molecules. Struct. Chem..

[22] Hegazy MA (2022). Effect of CuO and graphene on PTFE microfibers: Experimental and modeling approaches. Polymers.

[23] El-Mansy MAM, Ibrahim M, Soliman HS, Atef SM (2021). FT-IR, molecular structure and nonlinear optical properties of 2-(pyranoquinolin-4-yl)malononitrile (PQMN): A DFT approach. Biointerface Res. Appl. Chem..

[24] Omar A (2022). Enhancing the optical properties of chitosan, carboxymethyl cellulose, sodium alginate modified with nano metal oxide and graphene oxide. Opt. Quant. Electron..

[25] Joseph K, Jolley K, Smith R (2015). Iron phosphate glasses: Structure determination and displacement energy thresholds, using a fixed charge potential model. J. Non-Cryst. Solids.

[26] Gaussian 09, Revision C.01, Frisch M. *et al.*, Gaussian, Inc., Wallingford CT, **2010**.

[27] Becke AD (1993). Density-functional thermochemistry. III. The role of exact exchange. J. Chem. Phys..

[28] Lee C, Yang W, Parr RG (1988). Development of the Colle-Salvetti correlation-energy formula into a functional of the electron density. Phys. Rev. B Condens. Matter..

[29] Miehlich B, Savin A, Stoll H, Preuss H (1989). Results obtained with the correlation energy density functionals of becke and Lee, Yang and Parr. Chem. Phys. Lett..

[30] O'Boyle NM, Tenderholt AL, Langner KM (2008). cclib: A library for package-independent computational chemistry algorithms. J. Comp. Chem..

[31] Tohamy KM, Abd El Sameea N, Soliman IE, Tiama TM (2012). Glass-ionomer cement SiO_2_, Al_2_O_3_, Na_2_O, CaO, P_2_O_5_, F- containing alternative additive of Zn and Sr prepared by sol–gel method. Egypt. J. Biophys. Biomed. Eng..

[32] Turker H, Yıldırım AB, Karakaş FP (2009). Sensitivity of bacteria isolated from fish to some medicinal plants. Turkish J. Fish. Aquat. Sci..

[33] Mabkhot YN (2016). Synthesis, molecular structure optimization, and cytotoxicity assay of a novel 2-acetyl-3-amino-5-[(2-oxopropyl)sulfanyl]-4-cyanothiophene. Molecules.

[34] Southam HM, Butler JA, Chapman JA, Poole RK, Poole RK (2017). The microbiology of ruthenium complexes. Advances in Microbial Physiology.

[35] Li W, Thian ES, Wang M, Wang Z, Ren L (2021). Surface design for antibacterial materials: From fundamentals to advanced strategies. Adv. Sci..

[36] Lemire J, Harrison J, Turner R (2013). Antimicrobial activity of metals: Mechanisms, molecular targets and applications. Nat. Rev. Microbiol..

[37] Bouarab-Chibane L (2019). Antibacterial properties of polyphenols: Characterization and QSAR (quantitative structure-activity relationship) models. Front. Microbiol..

[38] Durka K, Kamiński R, Luliński S, Serwatowski J, Woźniak K (2010). On the nature of the B…N interaction and the conformational flexibility of arylboronic azaesters. Phys. Chem. Chem. Phys..

[39] Rush JD, Lan J, Koppenol WH (1987). Effect of a dipole moment on the ionic strength dependence of electron-transfer reactions of cytochrome *c*. J. Am. Chem. Soc..

[40] Rezaei-Sameti M, Jukar NJ (2017). A computational study of nitramide adsorption on the electrical properties of pristine and C-replaced boron nitride nanosheet. J. Nanostruct. Chem..

[41] Refaat A, Youness RA, Taha MA, Ibrahim M (2017). Effect of zinc oxide on the electronic properties of carbonated hydroxyapatite. J. Mol. Struct..

[42] Zhang L, Ding Y, Povey M, York D (2008). ZnO nanofluids—A potential antibacterial agent. Prog. Nat. Sci..

[43] Sirelkhatim A (2015). Review on zinc oxide nanoparticles: Antibacterial activity and toxicity mechanism. Nano-Micro Lett..

[44] Drago L, Toscano M, Bottagisio M (2018). Recent evidence on bioactive glass antimicrobial and antibiofilm activity: A mini-review. Materials.

[45] Kim DH, Marbois BN, Faull KF, Eckhert CD (2003). Esterification of borate with NAD+ and NADH as studied by electrospray ionization mass spectrometry and 11B NMR spectroscopy. J. Mass Spectrom..

